# How to build your own island

**DOI:** 10.7554/eLife.04779

**Published:** 2014-10-21

**Authors:** Colum Walsh, Avinash Thakur

**Affiliations:** 1 **Colum Walsh** is at the Biomedical Sciences Research Institute, University of Ulster, Coleraine, United Kingdomcp.walsh@ulster.ac.uk; 2 **Avinash Thakur** is at the Biomedical Sciences Research Institute, University of Ulster, Coleraine, United Kingdomthakur-a@email.ulster.ac.uk

**Keywords:** DNA methylation, epigenetics, high throughput genome editing, CpG islands, histone modifications, bivalent chromatin, human, mouse

## Abstract

Inserting artificially-generated ‘DNA islands’ into a genome has shed new light on why some DNA sequences are methylated and others are not.

**Related research articles** Wachter E, Quante T, Merusi C, Arczewska A, Stewart F, Webb S, Bird A. 2014. Synthetic CpG islands reveal DNA sequence determinants of chromatin structure. *eLife* **3**:e03397. doi: 10.7554/eLife.03397

Krebs AR, Dessus-Babus S, Burger L, Schübeler D. 2014. High-throughput engineering of a mammalian genome reveals building principles of methylation states at CG rich regions. *eLife* **3**:e04094. doi: 10.7554/eLife.04094

**Image** Which features of CpG islands (blue) mark them as different from the rest of the genome (red)?

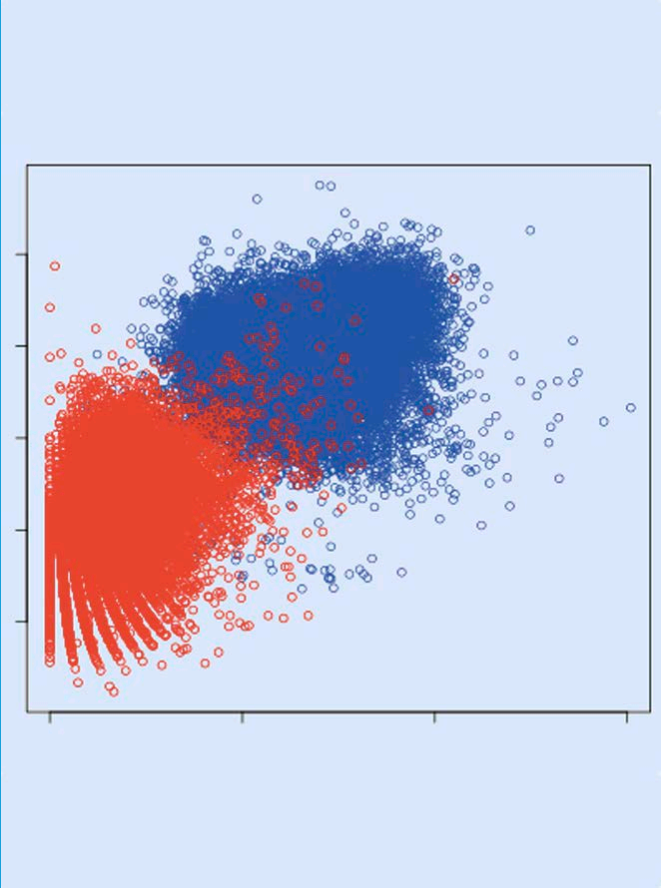

Navigating your way through the sea of information that is contained in the DNA of a genome can be a daunting task. The human genome, for example, contains about 3 billion base pairs and encodes around 20,000 genes. It is fortunate, therefore, that this sea is punctuated by ‘islands’ that mark the locations of important features. CpG islands, for example, mark the start of genes so reliably that they were used to identify genes in the pre-genomics era, before sequence information was so readily available. Despite this, we still do not fully understand why such islands are associated with essential DNA features, or which properties of these islands are crucial to their function. Now in *eLife*, two independent teams—one led by Adrian Bird, the other led by Dirk Schübeler—have shed more light on these enigmatic elements by taking advantage of recent advances in recombinant DNA technology.

While the four bases in DNA—A, C, T and G—are found in almost equal numbers in most DNA molecules, particular combinations of two bases can be more common in certain stretches of DNA than others. In particular, C is rarely followed by G (written as CpG); however, early research showed there are ‘islands’ where these CpG sites are common (Bird, 1980). Furthermore, these islands also have more Gs and Cs overall than the rest of the genome. This G + C enrichment does not always correlate with frequency of CpG sites, suggesting that the two properties should be considered separately.

CpG islands were also discovered to be important for transmitting epigenetic marks (that is, for transmitting heritable information that does not depend on the DNA itself; Mohandas et al., 1981; Li et al., 1993). A CpG site is recognised by enzymes that add a chemical tag called a methyl group to the C base. The methylation of the DNA in CpG islands inactivates nearby genes. Moreover, these epigenetic marks can be recreated whenever DNA is copied, and can therefore be passed on to new cells at each cell division. It is also known that some proteins bind specifically to methylated islands, and others to unmethylated islands (Nan et al., 1993; Lee et al., 2001). Both of these groups of binding proteins then recruit enzymes to the island. The enzymes in turn add methyl groups to the histone proteins, which help to package DNA in the cell. Different kinds of histone modifications can either activate or inactivate the nearby genes, and can also be passed on to new cells.

The Bird and Schübeler labs have taken advantage of recently developed ‘recombineering’ approaches which use high-throughput DNA technology to allow the efficient insertion of different DNA fragments into the same site in a genome (see Figure 1A). Bird and colleagues in Edinburgh and Dresden—including Elisabeth Wachter as first author—generated an artificial CpG island that resembled naturally-occurring ones in terms of CpG frequency and G + C density (Wachter et al., 2014). After this island was inserted into a region of the genome that contained relatively few genes, the DNA on the island was unmethylated (Figure 1B). Furthermore, the artificial island was able to recruit histone-modifying enzymes, as shown by the accumulation of two kinds of chemical marks on the histones. These histone marks are normally associated with promoters in embryonic stem cells: one is found on active promoters, and the other is associated with inactive promoters. The artificial island therefore seemed ‘poised’ for either activation or repression, even though there were no genes nearby.

**Figure 1.**
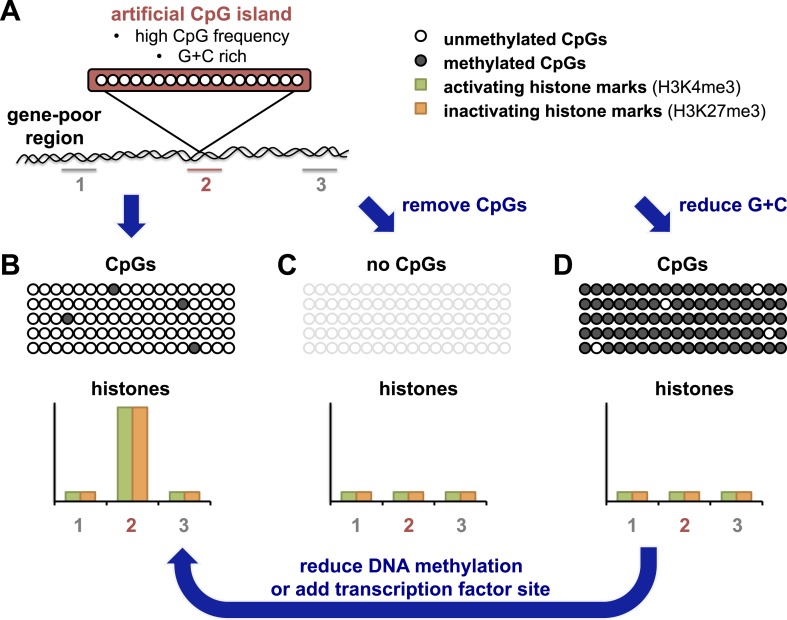
An artificial CpG island reveals its secrets. (**A**) Wachter et al. inserted stretches of DNA that were G + C rich, and also had a high frequency of CpG sites, into an area of the genome that is devoid of genes (‘gene-poor region’). Histones associated with the DNA were assayed for histone marks: the number 2 indicates histones associated with the artificial CpG island; 1 and 3 indicate histones not associated with the island. (**B**) The CpG sites in these artificial islands remained unmethylated and recruited both activating and inactivating histone marks (labelled H3K4me3 and H3K27me3 respectively). The rows of open or filled circles represent unmethylated or methylated CpG sites; and the graph represents the frequency of each histone mark in different regions, in and around the artificial island. (**C**) Removal of CpG sites from the artificial island prevented the accumulation of both types of histone mark. (**D**) Decreasing the G + C content caused CpG sites to be over-methylated, which blocked histone modification. However, this could be overcome, at least in part, by either preventing CpG site methylation to begin with, or (as revealed by Krebs et al.) by adding a strong binding site for a transcription factor into the island which could drive its demethylation (arrow).

Wachter et al. then resynthesized versions of the same artificial CpG island, but with either fewer CpG sites and the same number of Gs and Cs, or vice versa. By altering these two features separately, they could address the roles of each of these features for the first time. When CpG sites were essentially removed, but G + C content remained the same, histone modifications were absent (Figure 1C). This suggests that a high frequency of CpG sites is what leads to the ‘poised’ state described above.

When Wachter et al. tested this and reduced the G + C content of the artificial island, but left the CpG frequency unchanged, they found unexpectedly that the island also showed no histone modifications (Figure 1D). However, the DNA of this ‘G + C poor’ island was methylated, which seemed to block modifications to histones. This idea was later confirmed by making artificial CpG islands in cells that cannot target any new DNA for methylation (Figure 1D, arrow). In this background, the G + C poor island could once more recruit both activating and inactivating histone modifications, albeit at lower levels. These results highlight the importance of a high G + C content in protecting the DNA of CpG islands from being methylated.

The Schübeler lab in Basel has previously used a limited recombineering approach to begin to identify DNA sequences that can recreate their normal methylation pattern even when they moved them to a new location in the genome (Lienert et al., 2011). Now Schübler and colleagues—including Arnaud Krebs as first author—have used a similar approach but with a more extensive collection of DNA fragment sizes and types (Krebs et al., 2014). They compared the methylation of each fragment in its original position and in its ‘transplanted’ location. The findings of Kreb et al. largely matched those of Wachter et al., and show that DNA fragments or islands with a high CpG frequency retain a non-methylated state, whereas other fragments show more variation in their methylation patterns. Krebs et al. also observed that CpG islands with binding sites for transcription factors (proteins that help to switch gene expression on or off) were more likely to be unmethylated. Furthermore, Krebs et al. could drive the demethylation of an artificial island with an intermediate CpG density by inserting a binding site for one such transcription factor (called REST) into it (Figure 1D, arrow). These results suggest that proteins that strongly activate gene expression can influence the final methylation state that is acquired.

Wachter et al. and Krebs et al. both highlight the ability of the primary DNA sequence to program the default epigenetic state, and show that this can be further influenced by transcription factors for some types of genomic island. They also indicate that proteins that bind to areas of high G + C content may be crucial for protection against DNA methylation. However, it remains unclear if proteins may also exist that recognise A + T rich regions and recruit DNA-methylating enzymes to them. Furthermore, the component that recruits the enzymes that add the inactivating marks to the histones in areas of high CpG frequency is also currently unknown. As such, there is still a lot to learn about island-binding proteins. However, research in this field looks promising and it will undoubtedly help guide us through the sea of information that is contained within our own genome, and the genomes of other species.
